# Abortion Ban and the Next Generation’s Family Formation Decisions: Evidence from Romania

**DOI:** 10.1007/s10680-026-09768-8

**Published:** 2026-03-02

**Authors:** Selin Köksal, Nicoletta Balbo, Francesco C. Billari

**Affiliations:** 1 Department of Population Health, London School of Hygiene and Tropical Medicine, London, UK; 2 Department of Social and Political Sciences and Dondena Centre for Research on Social Dynamics and Public Policy, Bocconi University, Milan, Italy

**Keywords:** Abortion ban, Census data, Family formation, Generations and Gender Survey, Romania, Regression discontinuity design

## Abstract

This study examines how the introduction of an abortion ban can shape the next generation’s family formation trajectories, and whether the influence of the ban varies by gender and parental socioeconomic status (SES). Focusing on Romania’s abortion ban period under the Ceaușescu regime, we investigate changes in the probability of being married, the age at leaving the parental home and at first marriage, and spousal age characteristics. Using two complementary datasets, the 2011 Romanian census and the 2005 Generations and Gender Survey, and employing a regression discontinuity design, we find that compared to their counterparts born before the abortion ban, women born under the ban regime left their parental home later, formed their first marriage later, and had a lower probability of being married. This postponement effect was mainly driven by women with lower parental SES. Conversely, we do not observe significant changes in men’s family formation outcomes, except for a higher likelihood of marriage among men with higher parental SES who were born after the ban. The spousal age difference was smaller for both women and men born during the abortion ban. Findings show that abortion restrictions can have lasting effects not only on the individuals who are directly affected by them, but also on the next generation’s family formation trajectories.

**Supplementary Information:**

The online version contains supplementary material available at 10.1007/s10680-026-09768-8.

## Introduction

Before December 1966, Romania had one of the most liberal abortion policies in Europe, allowing free and safe access to abortion within the first trimester of pregnancy (Pop-Eleches, 2006). During the rule of Nicolae Ceauşescu, the so-called *Decree 770* banned, without prior warning, abortion for all women except for women who were older than age 45, had more than four children, had at-risk pregnancies, or became pregnant due to rape or incest. This sudden enactment led to a boom in unintended and mistimed pregnancies, and to an immediate increase in the number of births, as abortion was widely used as a primary birth control method in Romania, with four abortions being recorded for every live birth in 1965 (Berelson, 1979). The number of births doubled from 1966 to 1967, and remained high during the following four years (Mureşan, 2008). From 1967 to 1971, Romania’s total fertility rate (TFR) of about 3.21 was approximately 50% higher than the average TFR of about 2.15 in five other Central and Eastern European countries of the Soviet bloc: Bulgaria, Czechoslovakia, the German Democratic Republic, Hungary, and Poland (Berelson, 1979). Until December 1989, abortion and contraceptive methods remained illegal in Romania. The subsequent abolition of the ban coincided with a renewed increase in the number of abortions and a decline in fertility (Mitrut & Wolff, 2011).

Policies targeting women’s reproductive rights have long-lasting effects not only on the individuals who are directly affected (Everett et al., 2024), but also on their children, i.e., the “next generation.” In terms of the direct effects, legal access to abortion services helps women better control the timing of their childbearing, which improves their children’s economic opportunities (Ananat & Hungerman, 2012; Bailey et al., 2018; Mølland, 2016). Conversely, abortion bans may shape the next generation’s life course by increasing the size of the cohort into which they are born. Members of these relatively large cohorts tend to postpone the timing of union formation and childbearing, and subsequently have fewer children (Easterlin, 1978, 1987). Furthermore, by increasing the proportion of “unwanted” births in a population, abortion bans lead to children being raised under disadvantageous economic circumstances (Bitler & Zavodny, 2004) and receiving lower parental investments. These factors are associated with an array of negative outcomes, including poorer health (Singh et al., 2012) and lower educational attainment (Rastogi & Sharma, 2022).

In this paper, we extend the literature focused on the intergenerational effects of the Romanian abortion ban—e.g., on educational attainment, labor market outcomes (Pop-Eleches, 2006), cognitive abilities (Botezat & Levels, 2017), fertility (Gutierrez, 2022), and crime (Hjalmarsson et al., 2021)—by investigating the effects of the ban on the next generation’s family formation decisions, which have so far remained unexplored. Investigating how restricting parents’ access to abortion may shape the next generation’s family life trajectories enriches our understanding of the long-term and persisting effects of reproductive rights restrictions. In doing so, we underscore the relevance of taking a long-term and intergenerational perspective when seeking to unravel the complexity of family formation dynamics.

As our first contribution, we assess whether the increase in cohort size induced by the abortion ban shaped the timing and the probability of union formation among women and men of the next generation. We also explore whether the abortion ban changed when children left the parental home, given that Romanians typically do not leave the parental home until they marry, or even later (Mureşan, 2007). The gendered effect of the Romanian abortion ban has not been studied in prior work, even though both cohort size and unwantedness mechanisms may have different implications for the various outcomes of women and men. Moreover, we examine whether these relationships vary by parental socioeconomic status. The stratification perspective is particularly relevant when studying reproductive rights because it has been shown that the decision to terminate a pregnancy and access to abortion services and oral contraceptives are socially stratified (England, 2016). In 1960s Romania, educated women living in urban areas were more likely than other groups to use abortion services and oral contraceptives before the ban (Pop-Eleches, 2006). At the same time, less-educated women had a greater excess fertility burden due to the abortion ban than their better-educated and high-income counterparts, who had better sexual health knowledge (Rada, 2014) and easier access to external options for abortion and contraception under the restrictive regime (Pop-Eleches, 2010).

From a societal point of view, this study provides further evidence of the long-lasting and socially stratified consequences of policies restricting abortion access, which are being discussed in today’s political debates.

From an empirical perspective, this work advances the literature by better isolating potential confounding factors associated with being born in a relatively large cohort and life course outcomes. By employing a regression discontinuity design, which leverages an unexpected ban on abortion, we compare the family formation decisions of cohorts who were born just a few years before and after the abortion ban—i.e., members of a generation who underwent key life course transitions within a similar social, political, and economic context.

Lastly, we use data from two complementary sources: the Romanian census and the Generations and Gender Survey (GGS). While prior literature exploring the intergenerational effects of the Romanian abortion ban used census data (Gutierrez, 2022; Pop-Eleches, 2006), these data do not provide information on individuals’ parental background, and are of limited use for directly addressing the issue of selection into abortion services. We thus take advantage of the GGS, which includes retrospective information on the respondents’ parental background, and enables us to uncover new evidence on the socially stratified consequences of the abortion ban. The GGS also allows us to conduct a comprehensive analysis of key life course events, including leaving the parental home and union formation among both men and women born after the abortion ban.

## Theoretical Overview

### Abortion Ban in Romania

Following the slowdown in birth rates and population growth in 1960, Ceaușescu’s government banned abortion and heavily restricted all means of modern contraceptive methods in December 1966. Considered one of the world’s most rigidly enforced pronatalist population policies, the ban not only restricted access to abortion and contraception but also heavily monitored women’s reproductive lives through mandatory gynecological exams and additional income taxes for married couples who did not have a child within the first two years of their marriage, unless there was medical certification for infertility (Hord et al., 1991). Moreover, illegal abortion allegations were closely investigated by the Romanian state security police amd self-induced abortions and medical staff performing illegal abortions were subjected to imprisonment (Hord et al., 1991).

**Fig. 1:**
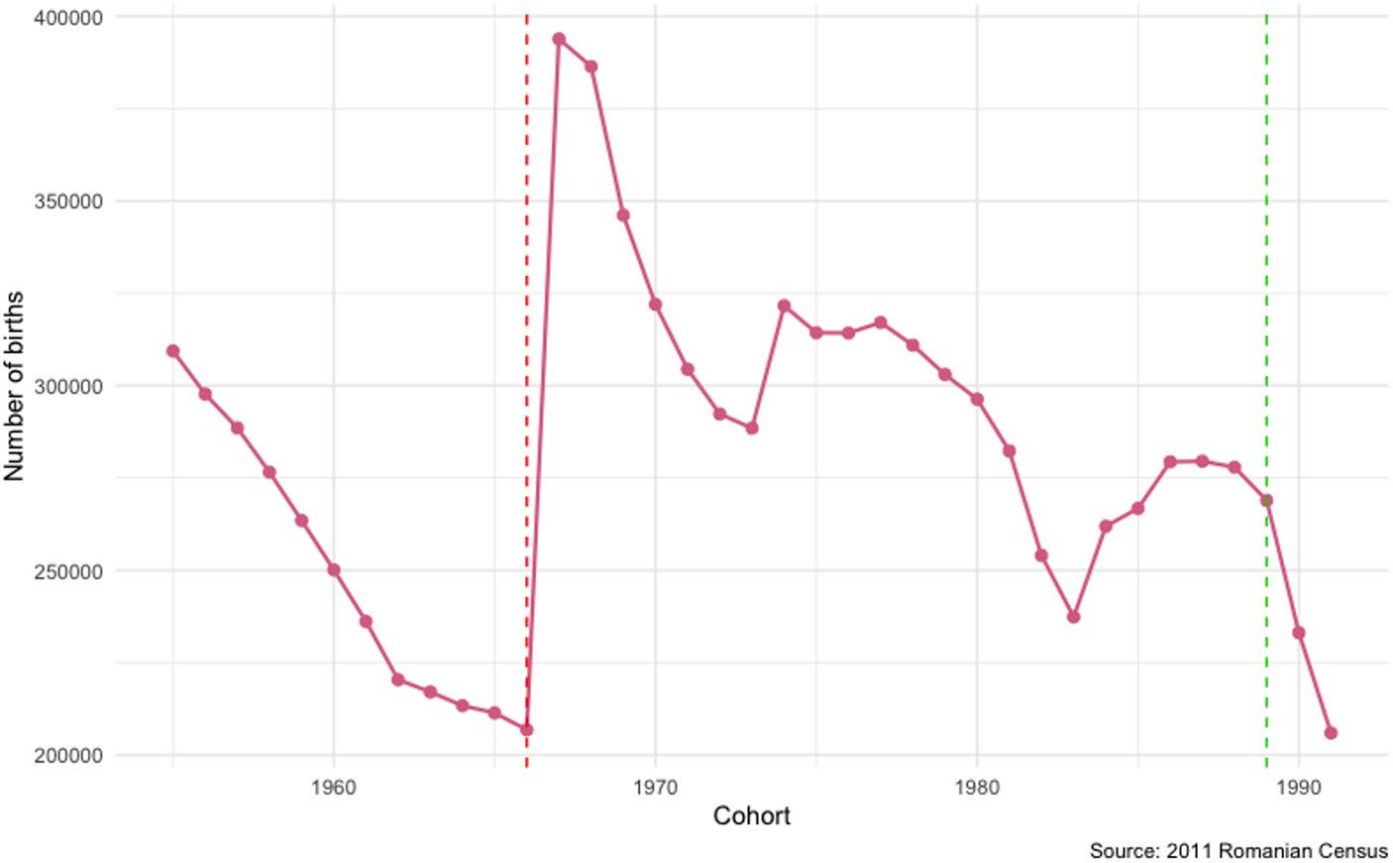
Number of births between 1955 and 1995 in Romania

The most immediate consequence of such a rigid abortion ban, as displayed in Fig. 1, was that the number of births doubled from 1966 to 1967 and remained elevated over the following four years. This generated unusually crowded cohorts for those who were born in the first years of the abortion ban as compared to those who were born immediately before., we expect the ban to influence family formation decisions of those born during this period in two ways. First, we suggest that cohort size links an abortion ban to family formation decisions, as it is directly connected to marriage market dynamics and economic uncertainty. Larger cohorts face increased competition in both marriage and labor markets, which may alter the timing and likelihood of partnership formation. Second, as the abrupt enforcement of the ban led to a substantial number of unwanted and mistimed pregnancies being carried to term, being born during the abortion ban period can also be linked to family formation decisions through the unwantedness mechanism.

As we acknowledge in the next subsections, Romania’s political regime and the economic and social life at the time had specific characteristics and incentives both on the marriage market (i.e., “celibate” tax and high age hypergamy) as well as the labor market (i.e., free universal education and low unemployment) that likely shaped the abortion ban effects under study. Consequently, we restricted our analyses to the cohorts born right before and after the introduction of the ban, who experienced the transition to adulthood still under the same political regime. In this way, we aim to reduce the risk of confounding the abortion ban effects with the ramifications of other significant societal changes following the fall of the regime in 1989.

### Cohort Size

#### Cohort Size and Uncertainty

According to the Easterlin hypothesis (Easterlin, 1978, 1987), individuals born into larger birth cohorts face more crowded labor markets and fiercer competition for jobs, leading to lower real wages. As a result of this economic adversity, individuals born into larger cohorts might delay major life events, such as leaving the parental home, getting married, and having children (Pampel & Peters, 1995). Evidence on the effects of Romania’s abortion ban shows that due to crowding in schools, children born after the abortion ban had poorer educational and labor market outcomes compared to their counterparts born before the ban (Pop-Eleches, 2006). Economic uncertainty, which is often linked with adverse educational and labor market outcomes, may result in the postponement of union formation or a lower likelihood of marriage (Pampel & Peters, 1995).

The empirical support for the Easterlin hypothesis is rather mixed. While it was empirically validated in the U.S., it has not gained support in the European context mainly due to differences in institutional factors such as unemployment benefits, housing subsidies, and family allowances, which can mitigate the economic effects of large cohort sizes. In contrast to this hypothesis, Carlson (1992) suggested that in the state-controlled economies of socialist Eastern Europe, which had virtually full employment, firms were always willing to hire more workers (Kornai et al., 1982), leading large cohorts to experience economic booms that improved the provision of goods and services and enhanced overall economic well-being. Indeed, the reproduction of the labor force was the primary motivation for Ceaușescu’s pronatalist policies in socialist Romania. Moreover, the labor market structures and cultural norms surrounding work and family influenced how economic uncertainty affected family formation (Özcan et al., 2010). For instance, in the former socialist bloc, economic security was not considered a prerequisite for entering a union or starting a family. Instead, forming a family and building a professional career were perceived as parallel endeavors that could be pursued simultaneously (Bernardi et al., 2008). Thus, while cohorts born after the abortion ban experienced worse educational and labor market outcomes, these disadvantages may not have translated into delayed or foregone marriage as the Easterlin hypothesis would predict in capitalist contexts.

The ban could affect the timing of family formation through other channels, particularly housing constraints. In socialist Romania, leaving the parental home was closely tied to union formation, with many couples initially residing with the husband’s parents due to severe housing shortages (Mureşan, 2007). Both women and men typically left their parental homes at the time of marriage, with women often moving into their husband’s or his parents’ household. The increased demand for housing from the larger cohorts born after the ban, combined with the rigid state allocation system and lack of infrastructure to meet housing demand (Kligman, 1998), likely created delays in securing independent living arrangements and thus delayed the timing of both departure from parental home and marriage.

Overall, we expect that both women and men who were born after the abortion ban experienced delays in the timing of leaving their parental home and union formation due to crowding effects, particularly housing shortages (Hypothesis 1). However, given that marriage was nearly universal in socialist Romania and economic security was not a prerequisite for family formation, we expect no changes in the ultimate probability of marriage both for men and women. (Hypothesis 2).

#### Cohort Size and Marriage Market

In the context of socialist Romania, marriage and parenthood were highly valued and perceived as moral obligations and sources of personal fulfillment (Kligman, 1998). Marriage was nearly universal and early marriage was promoted by the collection of a “celibate” tax from unmarried individuals (Nadolu et al., 2007). Furthermore, the Romanian marriage market was characterized by age hypergamy whereby women typically partnered with older men, with an average age difference of three years (Mureşan, 2007).

After the enactment of the abortion ban, the sudden increase in cohort size disrupted the balance of the marriage market in gender-specific ways. Finding an eligible partner may have become more challenging, particularly for younger women born after the ban, as they outnumbered older men from the smaller pre-ban cohorts. Given the prevailing norm of age hypergamy, women from the enlarged post-ban cohorts faced a relative shortage of potential partners in the traditionally preferred older age groups. Indeed, descriptive evidence from Romania suggests that a higher proportion of women in the cohorts born immediately after the abortion ban formed unions with same-age or younger partners than in the older cohorts (Cutler, 2012), indicating an adaptation to this marriage market squeeze.

Conversely, for men, the pool of eligible partners expanded substantially as the younger cohorts born under the abortion ban regime were significantly more numerous than the cohorts not exposed to the abortion ban. A marriage squeeze against women (when eligible women outnumber eligible men) usually has a small effect on the probability of marriage while it is expected to significant effects on age at marriage by lowering the timing of entry to marriage for men (Schoen, 1983). Considering the pro-family and pro-natalist context of socialist Romania, marriages was incentivized by celibate taxes and housing provision to marriage couples (Kligman, 1998) it is plausible that men’s probability of marriage also increased. Thus, the change in cohort size induced by the abortion ban may allow men to form unions earlier and may even increase their probability of marriage given the increased number of younger potential partners.

Based on this, we hypothesize that women born after the abortion ban experienced delays in leaving the parental home and union formation and had a lower probability of ever marrying compared to their counterparts born before the ban, due to the marriage market squeeze created by age hypergamy norms. (Hypothesis 3a). Additionally, among post-ban married women, we expect a decline in the spousal age gap compared to women born before the ban as the shortage of older male partners led women to form unions with same-age or younger partners (Hypothesis 3b).

For men, we expect to find that those born after the ban entered their first union earlier than men born before the ban. Additionally, we hypothesize that post-ban men had a higher probability of ever marrying than men born before the ban, due to their expanded pool of younger potential partners (Hypothesis 4a). Relatedly, we expect little to no change in the spousal age gap compared to men born before the ban, as the increased number of younger partners allowed men to maintain conventional age difference (Hypothesis 4b).

### The Role of Parental Support

#### Parental SES

By the 1980s, Romanians were experiencing declining purchasing power and deteriorating living conditions, which led to widespread economic hardship (Kligman, 1998). The surge in births, coupled with a lack of infrastructure and financial resources, burdened the school system and lowered the quality of public education (Kligman, 1998). Even though education was free at all levels, there were admission exams for high schools and universities, and affluent families often resorted to private tutoring to secure their children’s admission to prestigious schools (Kligman, 1998). Thus, parents’ socioeconomic background played a significant role in shaping children’s educational and labor market outcomes in Romania and in other Eastern European countries (Iannelli & Smyth, 2008).

Under the socialist regime, a housing shortage created significant challenges for young couples looking for a place to live, resulting in many of them residing with their parents, usually the parents of the male partner. This living arrangement sometimes continued until the birth of the first child, at which point the couple was allocated an individual dwelling by the government (Castiglioni et al., 2016). This pattern was less prevalent among high-SES couples or couples with greater financial resources (Mureşan, 2007). Higher parental SES and home ownership have been shown to facilitate the transition to marriage, particularly for men (Lloyd & South, 1996). High-SES parents can use their financial resources to support their children’s new household by contributing to marriage expenses and the purchase of a home (Mulder et al., 2006). Thus, parental resources could buffer children from both the cohort crowding effects (through access to better education and housing) and enable them to form unions earlier by providing the necessary material foundation for marriage.

#### Unwantedness Mechanism

The surge in unwanted pregnancies during the abortion ban period led to an increase in child abandonment and institutionalization. Despite pursuing a stringent pro-natalist agenda, the Romanian government failed to provide the necessary economic resources and health assistance to support families. Although unwanted pregnancies occurred across all socioeconomic strata, the consequences differed dramatically by parental SES. While the immediate impact of the abortion ban was more pronounced among urban and high-income women, as they were using abortion services more frequently due to their lower fertility intentions (Pop-Eleches, 2006), the ban took a higher toll on poorer women who lacked the means to seek abortions elsewhere and the resources to care for another child (Kligman, 1998; Pop-Eleches, 2010).

Child abandonment became a coping mechanism, especially for low-income families. Unofficial estimates suggest that approximately 150,000 to 200,000 children were abandoned or institutionalized during the abortion ban period (Stephenson et al., 1992). The orphanages were characterized by poor hygiene and sanitation conditions as well as severe understaffing, which significantly affected the abandoned children’s educational and health trajectories (Morrison, 2004). The rates of developmental disabilities and infectious diseases were particularly high among institutionalized children.

Even among children who were not abandoned, experiencing an unwanted pregnancy can be challenging as parents may not have the optimal economic, social, and health conditions for raising a child, which could have long-term implications for the child’s life course. Unwanted pregnancies are associated with delayed or irregular prenatal care (Joyce & Grossman, 1990), reduced folic acid intake, and a higher risk of tobacco use during pregnancy (Cheng et al., 2009). Consequently, unwanted pregnancies carry a higher risk of pre-term labor and low birthweight (Bruckner & Catalano, 2018), which are key predictors of long-term inequality throughout the life course (Aizer & Currie, 2014). Furthermore, children born from unwanted pregnancies are less likely to be breastfed (Kost & Lindberg, 2015), to receive all recommended vaccinations (Singh et al., 2012), and, in the case of daughters, to graduate from high school and attend college (Rastogi & Sharma, 2022). In the context of Romania, children born after the lifting of the abortion ban had a lower risk of low birth weight (below 3 kg) compared to those born during the ban (Mitrut & Wolff, 2011).

As cumulative inequality theory suggests, negative experiences in childhood play a crucial role in shaping adulthood lifestyles, resources, and decisions (Ferraro et al., 2009). Accordingly, early life health disadvantages are linked with greater social isolation (Lebenbaum et al., 2024), public and self-stigmatization (Kroeger, 2021), and poorer mental and physical health in both adolescence (McLaughlin et al., 2012) and adulthood (Ferraro et al., 2016), which may, in turn, shape individuals’ family formation decisions (Evensen & Lyngstad, 2020).

We hypothesize that the delays in family formation trajectories and declining probability of marriage among women born after the ban were significantly larger for women from lower parental socioeconomic backgrounds compared to women from higher parental SES, due to unwantedness effect and the lack of parental resources to buffer against crowding effects (Hypothesis 5).

We hypothesize that the anticipatory effect in marriage timing and increased probability of union formation among men born after the abortion ban was significantly stronger for men with higher parental SES compared to men with lower parental SES, as high-SES men had access to parental resources necessary to actualize favorable marriage market conditions (Hypothesis 6).

## Data

### Analytical Sample

Our analyses draw on two data sources. The first is the Romanian census of 2011, which was conducted by the Romanian National Institute of Statistics and made available through IPUMS-International. This census includes a randomly selected 10% sample of the entire population among whom detailed information was collected on the age, education, and marital status of each household member, and, specifically among women, on the age at marriage and the number of ever-born children. In our analyses, we include respondents born in Romania (14,251 observations, 99.3% of the total sample).

To complement the census data, we use data from wave 1 of the Romanian Generations and Gender Survey (hereafter GGS). The Romanian GGS is a nationally representative survey that collected retrospective data on the quantity and the timing of fertility events and marriage and cohabitation, and on several socioeconomic indicators, among individuals aged 18 to 80 in 2005. Given the decree’s direct impact on Romanian women residing in Romania, we exclude respondents whose mothers did not have Romanian citizenship (79 observations, 0.6% of the sample) or who were born abroad (15 observations, 0.1% of the sample).

### Treatment Status

Before the enactment of the abortion ban, Romanian women could legally terminate unintended pregnancies within the first trimester. However, from October 1966 onward, pregnancies were subjected to restrictions, as their first trimester overlapped with the introduction of the abortion ban in December 1966. Consequently, mothers of individuals conceived during this period were unable to legally terminate their pregnancies. Therefore, it is appropriate to consider the individuals conceived from October 1966 onward as directly affected, or “treated,” by the abortion ban. Given the lack of data on the conception date in both the census and the GGS, we approximate the date of conception as nine months before the birth date, establishing June 1967 as the cut-off point for determining treatment status. Individuals born after June 1967 are classified as affected by the ban (treated), while those born before this month are in the control group. This cut-off point aligns with those used in previous studies on the Romanian abortion ban (Pop-Eleches, 2006).

### Outcome Variables

In terms of outcome variables, we use census data to examine marital status (1 = married, 0 = other), women’s age at first marriage, and the age difference between married couples living in the same household at the time of the survey. The age difference variable corresponds to age difference between the male and the female partner. We focus on the woman’s age at first marriage, as this question was posed to female respondents only.

From the GGS data, we make use of the information on the age when individuals left their parental home, their age at first marriage, and their marital status at the time of the interview (1 = married, 0 = other).

### Moderating Factors

We examine whether the effect of abortion on the family formation trajectories of the next generation varied by parental SES. To do so, we rely on GGS data, which includes information on the educational attainment of respondents’ parents. Parental education is the only available parental SES measure within the GGS data. For simplicity, we define parental SES by considering the highest level of education attained by either parent. Respondents are classified as having low parental SES if the highest educational level obtained by either parent was a lower secondary degree (International Standard Classification of Education (hereafter ISCED) 2), and as having high parental SES if the highest educational level obtained by either parent was an upper secondary degree (ISCED 3).

## Empirical Strategy

Employing June 1967 as the cut-off point, we use a regression discontinuity (RD hereafter) to estimate how an abortion ban can shape the family formation decisions of individuals in the next generation.

Below we present the model used in the first step of the empirical analysis:

$$\:{Y}_{i}=\:{\beta\:}_{0}+\:{\beta\:}_{1}{PostBan}_{i}+f\left({x}_{i}\right)+{\epsilon\:}_{m}$$

$$\:\forall\:{x}_{i}\:\in\:(c-h,\:c+h)\:$$

where $\:{Y}_{i}$ is one of the outcome variables presented in the data section, $\:{PostBan}_{i}$ indicates the treatment status, $\:{x}_{i}\:$is the running variable (i.e., month and year of birth) and $\:h$ is the bandwidth around the cut-off point $\:c$, and $\:{\epsilon\:}_{m}$ represents clustered standard errors at the month of birth level.

Our analysis of the abortion ban is performed using local linear regression. We have chosen an optimal bandwidth determined by a data-driven approach typically used within the RD framework. This strategy offers a robust alternative to the arbitrary bandwidth selection that has been previously applied in studies focusing on the Romanian abortion ban (Gutierrez, 2022; Hjalmarsson et al., 2021; Pop-Eleches, 2010), as it uses Mean Squared Error (MSE)-optimal bandwidth selection (Calonico et al., 2018) to determine the bandwidth in a way to allow efficient and accurate estimation of the effect of being born during the abortion ban. As a robustness check, we replicated our analyses by halving the optimal bandwidth. In all our RD analyses, we applied triangular kernel weights, which assign higher weights to observations closer to the cutoff date and gradually lower weight to observations further from the cutoff. This weighting approach ensures that our estimates prioritize comparisons between individuals born just before and after the abortion ban.

Besides these methodological considerations, the robustness check using narrower bandwidth helped us to mitigate the confounding effects of the dissolution of the Soviet Union in 1989, which led to the postponement of union formation mainly in response to the subsequent economic and political uncertainty (Sobotka et al., 2003). By restricting our analysis to cohorts born closer to the cutoff point and applying triangular kernel weighting, we reduce the likelihood that our estimates reflect the ramifications of significant societal changes following the fall of the regime in 1989 rather than direct effects of the abortion ban per se. Additionally, we include month of birth fixed effects to adjust for the seasonality in birth rates, and we cluster standard errors at the month of birth level to account for the unobserved characteristics of the individuals who were born in the same month (Lee and Card, 2008).

In the second step, we explore whether the abortion ban’s influence on individuals’ family formation decisions varied by parental socioeconomic status (SES). We investigate the effect of the abortion ban on the life course at the intersection of gender and parental SES by categorizing the analytical sample into four subsamples: (i) women with low parental SES, (ii) women with high parental SES, (iii) men with low parental SES, and (iv) men with high parental SES.

**Fig. 2:**
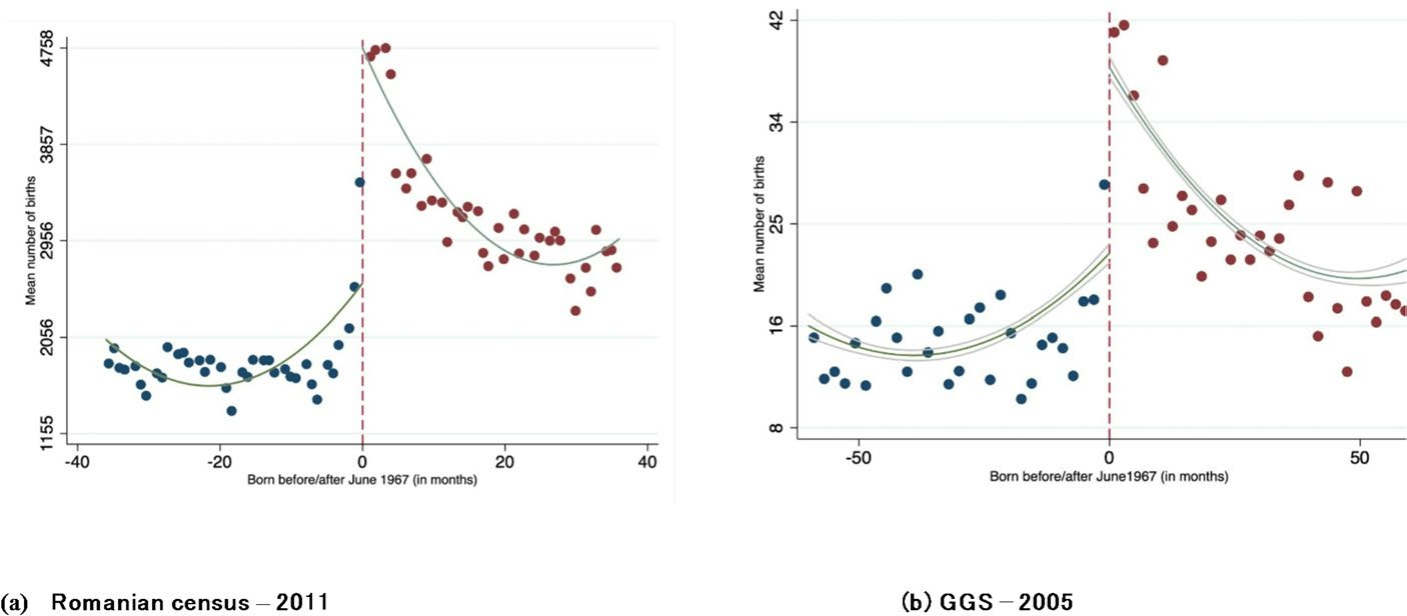
Mean number of births before and after the abortion ban by data source

For the abortion ban to create a plausibly exogenous discontinuity in the number of births, it must be assumed that individuals living in Romania at the time the abortion ban was imposed were not able to anticipate the decree and adjust their reproductive behavior accordingly. In essence, the likelihood of conception in September 1966 should not significantly differ from that in October 1966. The McCrary density check provides a formal test to validate the continuity of the running variable, (i.e., date of *conception*), around the designated cut-off point. However, both the census and the GGS dataset only record the date of *birth*. We thus retrospectively estimate the date of conception based on the date of birth, introducing a discontinuity in the running variable at the cut-off point. Consequently, if the enactment of the ban was unforeseen, we would expect to observe a significant increase in the number of births from May 1967 to June 1967. To test this assumption, we present the average number of births around the cut-off point in the census and the GGS data. Figure 2 displays a marked increase in births after June 1967, indicating that the abortion ban was indeed unexpected.

## Results

### Descriptive Evidence

Table 1 presents the summary statistics by gender of the outcome and the control variables, and their mean differences by the treatment variable. We provide summary statistics on the individuals who were born 50 months before and after June 1967 for the census data, and on those who were born 75 months before and after 1967 for the GGS data, as the estimated bandwidths in the RD analyses fall into these ranges.

**Table 1:** Descriptive statistics of the analytical sample

	Women				Men			
	Pre-ban	Post-ban	Dif.	Obs.	Pre-ban	Post-ban	Dif.	Obs.
	Mean	Mean			Mean	Mean		
Panel A: Census 2011								
Outcome variables								
Married	0.792	0.799	− 0.01	120,484	0.791	0.781	0.01	123,575
Age at first marriage	22.18	22.86	− 0.68**	87,330	–	–	–	–
Age difference	3.839	3.198	0.64**	80,733	3.526	3.017	0.51**	78,855
Panel B: GGS 2005								
Outcome variables								
Age at leaving home	19.9	19.89	0.01	1229	23.01	22.26	0.75*	1193
Married	0.84	0.83	0.01	1342	0.84	0.8	0.04*	1463
Age at first marriage	21.49	21.79	-0.3	1102	25.22	24.6	0.62*	1168
Age difference	3.65	3.37	0.28	1099	3.75	2.86	0.87**	1170
Moderating factors								
Parental SES (0 = low, 1 = high)	0.313	0.416	− 0.1**	1316	0.29	0.428	− 0.14**	1443

+*p* *<* 0.1, *p *<* 0.05, **p< 0.01

From the census data presented in Panel A, we can see that women born after the abortion ban had a higher age at first marriage (*p* < 0.01), while the spousal age difference was smaller for both men and women born after the ban compared to those born before (*p* < 0.01). The proportion married did not differ significantly between cohorts born before and after the ban.

Panel B, based on GGS data, shows no statistical differences between women born before and after the abortion ban in terms of age at leaving home age at first marriage, the proportion of married and spousal age differences. Men born after the abortion ban, however, left the parental home and formed their first marriages earlier, while the overall proportion of married men declined in post-ban cohorts (*p* < 0.5 for all outcomes). Moreover, the spousal age difference decreased for men born after the ban, meaning that they formed union with women closer to their age.

Additionally, parents who had children during the post-ban period were more likely to have an upper secondary degree than those who had children before the ban (*p* < 0.01). This latter finding applies for both women and men sample and aligns with Pop-Eleches(2006) study, which highlighted the SES gradient among parents of children born shortly after the abortion ban. We address this selection issue in our analyses using GGS data by first conducting a robustness check adjusting for parental SES, and then examining whether the intergenerational effects of the abortion ban on family outcomes for women and men varied by their parents’ SES.

### Findings Using Census Data

Table 2 documents the results obtained using census data. Accordingly, women born after the abortion ban were almost 2% point (− 0.016, *p* < 0.01) less likely to be married compared to their counterparts born after the ban. Conversely, among men, the probability of being married shows no significant change, regardless of whether they were born before or after the abortion ban. In terms of the timing of marriage, women born after the ban married 0.38 years later (0.38, *p* < 0.01) than those born before the ban.

**Table 2:** The effect of the abortion ban on marriage market outcomes using data from the Romanian census of 2011

	Women				Men			
	Linear RD	BW	Obs.	Mean	Linear RD	BW	Obs.	Mean
Married	− 0.016**	49	115,934	0.8	0	42	103,973	0.78
	(0.005)				(0.005)			
Age differences (men and women)	− 0.059	22	37,601	3.17	− 0.147**	36	57,792	3.17
	(0.079)				(0.058)			
Age at first marriage	0.380**	48	82,507	22.62				
	(0.071)							

Clustered standard errors in parentheses. +*p* *<* 0.1, *p *<* 0.05, **p *<* 0.01

In all specifications, month of birth is included as a control variable. BW: bandwidth; Obs.: number of observations

For men born after the abortion ban, the spousal age difference decreased by approximately 0.2 years (around 2.5 months, *p* < 0.05), while for women, there was no statistically significant change in the age difference between partners. These findings suggest that men born after the abortion ban were more likely to form unions with women closer to their own age than men born before the ban.

These results support Hypothesis 2 for men and Hypothesis 3a for women, highlighting the gendered effects of the abortion ban on marital timing and the age characteristics of spouses.

### Findings Using GGS Data

Table 3 presents the effects of the abortion ban on family formation trajectories by gender. Compared to their counterparts born before the abortion ban, women born after the ban left the parental home significantly later (1.3 years, *p* < 0.01) and delayed marriage (one year, *p* < 0.01). However, we do not observe any statistically significant difference in the likelihood of marriage between women born before and after the ban. Moreover, the age difference between male and female partner reduced by 0.8 years for women who were born after the abortion ban, but this difference is significant only at 10% significance level.

**Table 3:** The effect of the abortion ban on leaving the parental home and family formation by gender (GGS)

	Women				Men			
	Linear RD	BW	Obs.	Mean	Linear RD	BW	Obs.	Mean
Age at leaving home	1.313**	31	578	19.97	− 0.946	50	833	22.7
	(0.442)				(0.792)			
Married	− 0.0106	54	1026	0.84	0.083	43	893	0.82
	(0.0257)				(0.059)			
Age at first marriage	1.003**	56	878	21.63	− 0.727+	74	1143	24.84
	(0.329)				(0.434)			
Age differences (men-women)	− 0.806+	50	780	3.41	− 0.113	49	828	3.32
	(0.477)				(0.346)			

Clustered standard errors in parentheses. +*p<* 0.1, *p *<* 0.05, **p *<* 0.01. In all specifications, month of birth is included as a control variable. BW: bandwidth; Obs.: number of observations

Looking at men, we see that those who were born during the abortion ban period married almost 0.7 years earlier than their counterparts who were born before the abortion ban, but this effect was significant at the 10% level only. Moreover, men who were born after the ban left the parental home earlier and had a higher probability of forming a union than men who were born before the prohibition. As for spousal age differences, post-ban men showed smaller age gaps than pre-ban men. However, none of these coefficients were statistically different from zero. Overall, these findings lend partial support to Hypotheses 3a and 3b for women and partial support for Hypotheses 2 and 4b for men.

### Variation by Gender and Parental SES

Table 4 reports the results for women’s family formation decisions by their parental SES. Women from lower SES backgrounds who were born after the abortion ban left the parental home 1.7 (*p* < 0.01) years later and married one (*p* < 0.05) year later than their counterparts who were born before the abortion ban. Moreover, they were less likely to marry, although the difference was not statistically significant.

**Table 4:** The effect of the abortion ban on women’s decisions to leave the parental home and form a family by parental SES

	Women with lower parental SES				Women with higher parental SES			
	Linear RD	BW	Obs.	Mean	Linear RD	BW	Obs.	Mean
Age at leaving home	1.701**	33	389	19.13	0.309	71	414	21.39
	(0.528)				(0.707)			
Married	-0.036	73	807	0.85	0.123	47	344	0.81
	(0.043)				(0.086)			
Age at first marriage	1.014*	49	473	20.92	-0.370	64	350	22.76
	(0.414)				(0.851)			

Clustered standard errors in parentheses. +*p* *<* 0.1, *p *<* 0.05, **p *<* 0.01. In all specifications, month of birth is included as a control variable. BW: bandwidth; Obs.: number of observations

Conversely, we do not find any statistically significant effect of the abortion ban on the family formation decisions of women with a high SES background. Our findings thus support Hypothesis 5, which predicted that the delay in women’s family formation decisions would be driven by women with lower parental SES.

According to Table 5, men with higher parental SES who were born after the abortion ban are almost 18% points more likely to form a union (*p* < 0.01) compared to their counterparts born before the ban. While they also left their parental home and formed their first union earlier, this effect was not statistically different from zero. Conversely, we observe no statistically significant differences between low-SES men who were born before and after the ban in the timing of leaving the parental home and family formation and in the probability of marriage. Thus, the findings lend partial support to Hypothesis 6, which predicted that an increased probability of marriage among men who were born to higher-SES parents after the abortion ban.

**Table 5:** The effect of the abortion ban on men’s decisions to leave the parental home and form a family by parental SES

	Men with lower parental SES				Men with higher parental SES			
	Linear RD	BW	Obs.	Mean	Linear RD	BW	Obs.	Mean
Age at leaving home	− 0.78	56	573	22.33	− 0.315	47	282	23.53
	(0.914)				(1.056)			
Married	0.042	50	651	0.81	0.175**	34	250	0.84
	(0.06)				(0.055)			
Age at first marriage	− 0.566	38	426	24.68	− 0.974	50	299	24.93
	(0.492)				(0.653)			

Note: Clustered standard errors in parentheses. + *p* < 0.1, ** *p* < 0.05, ** *p* < 0.01. In all specifications, month of birth is included as a control variable. BW: bandwidth; Obs.: number of observations.

### Sensitivity Analyses

We conducted several sensitivity analyses, which are presented in Tables S1-S4 in the Supplementary Material. First, we replicated the census and the GGS analyses within the half of the optimal bandwidth – a common practice in RDD literature (Imbens & Lemieux, 2008). Table S1 shows that the results are very similar to those of the census analyses. Regarding the spousal age difference for women born after the abortion ban, we even estimate statistically significant and negative effect when we restricted our analyses to a smaller bandwidth (− 0.66 years, *p* < 0.01). Accordingly, the age difference between partners was smaller for women born during the abortion ban than for women born before the ban.

Table S2 also presents results qualitatively similar to those obtained in the GGS analyses. When using a narrower bandwidth, we find that the effect of the abortion ban on the delayed timing of leaving the parental home among women becomes statistically insignificant, and the effect’s magnitude drops considerably, although the direction remains the same. As for spousal differences, we estimate even larger and negative differences for women who were born after the abortion ban (− 1.4 years, *p* < 0.01). For the analyses by gender and parental SES, we could not replicate the models with a narrower bandwidth, as the number of observations was insufficient (*n* < 50) for a statistically powerful analysis.

Second, to address selection issues arising from the SES gradient among abortion seekers in pre-ban Romania, we re-ran the GGS analyses, adjusting separately for mother’s and father’s education (see Supplementary Material, Tables S3 and S4). The findings from the main analysis hold, both qualitatively and statistically.

## Discussion

Many governments actively engage in “strategic demography” by deploying fertility, mortality, or migration policies as key instruments of their national and international strategies (Teitelbaum, 2015). The Romanian abortion ban serves as a case study of pro-natalist policies aiming to boost labor supply, foster economic growth, and consolidate the regime’s future. Even though it was implemented to stimulate population growth, the abortion ban’s broader repercussions have echoed through the next generation’s life course. In this study, we investigated the impact of being born during the abortion ban on family formation transitions, employing data from the Romania census of 2011 and the Romanian GGS of 2005. While previous research examined the ban’s effects on various outcomes, the ways in which it shaped the next generation’s family formation decisions remained underexplored.

Findings from the census data show that women born during the abortion ban era experienced delays in first union formation compared to those born before the ban’s enactment. Additionally, these women had a lower probability of union formation, underscoring the ban’s long-lasting influence on marital dynamics. In contrast, no statistically significant differences in men’s probability of marriage were observed. In terms of spousal age differences, both men and women born during the abortion ban married partners closer to their own age.

To provide a more detailed picture of the family formation process in Romania, we complemented the census analyses with data from the Romanian GGS. Women whose parents were affected by the restrictive abortion regime experienced delays in leaving the parental home and in marrying compared to their counterparts born before the ban. Conversely, compared to their pre-ban counterparts, men born during the abortion ban period entered marriage slightly earlier without experiencing significant changes in their chances of being married.

These gender-specific outcomes, which were consistent across both the census and the GGS data, reflect the gendered dynamics of the Romanian marriage market, whereby younger women tend to marry older men (Mureşan, 2007). The increase in cohort size immediately after the enactment of the ban reduced women’s probability of finding an eligible partner, who was typically a man three years older. This shift was also reflected in the spousal age characteristics of individuals born during the abortion ban period. Accordingly, we found a smaller spousal age gap both for women and men who were born after the abortion ban compared to their counterparts who were born before.

Furthermore, the analyses indicated that the delays in leaving the parental home and union formation observed among women who were born after the abortion ban were mainly among those from families with lower levels of education. On the other hand, we found a higher likelihood of marriage among men born after the ban with higher parental SES backgrounds. This stratified effect of the abortion ban on women’s family formation decisions is consistent with the existing pattern of age hypergamy, which is particularly prevalent among low-SES families (Gustafson & Fransson, 2015). Additionally, as the abortion ban imposed greater reproductive and economic burdens on families with fewer resources (Kligman, 1998; Pop-Eleches, 2006, 2010) and exacerbated the challenges their children faced over the life course, it appears that only men who came from families with more resources were more likely to be married, despite the increase in the number of younger women.

The findings of this study contribute to the field of family sociology by underscoring the relevance of long-term and intergenerational perspectives in examining family formation trajectories. Moreover, considering the ongoing restrictions on abortion access in various contexts, this study provides further insights into the intergenerational repercussions of restrictive pronatalist policies. While the current restrictions on abortion services are primarily assessed in terms of their immediate impact on the affected generation, our study illustrates how past abortion bans can have spillover effects on the life course of subsequent generations. These effects could extend beyond maternal and infant health outcomes to significantly shape the family formation trajectories of these cohorts, an aspect that has remained largely unexplored until now. Finally, this study provides evidence that the long-term consequences of policies restricting abortion access are highly stratified by gender and SES.

The present study has several limitations. First, we were not able to disentangle the potential mechanisms listed in the theoretical overview section, especially due to the lack of data on parents’ pregnancy intentions and the status of institutionalized children. However, findings from the census data revealed a statistically meaningful decline in the age difference between partners among women born during the abortion ban period (see Table 2). This finding aligns with descriptive evidence for Romania indicating that a larger proportion of women married partners of a similar age or younger following the abortion decree (Cutler, 2012). While these gendered mating dynamics could explain the postponement of major life course events among women, distinguishing the relative contribution of the marriage market structure from that of being an “unwanted” child is challenging, as the two mechanisms mediate the effect in the same direction.

Second, the analyses conducted using the GGS had a smaller sample size. Working with a small sample led to the use of larger bandwidths, especially for the analyses examining the variations by gender and parental education level. While employing larger bandwidths in small samples increased precision, it also carried the risk of introducing bias into our estimations. To mitigate this concern, particularly for outcomes like the probability and the timing of marriage, we turned to analyses using census data. For instance, in line with the results obtained with the GGS data, the findings showed that the age at first marriage increased among women who were born after the abortion ban (see Table 1), albeit with a smaller magnitude (− 0.27 years, *p* < 0.01). The much larger number of observations in the census data – at least 40 times larger than that in the GGS – enabled us to estimate the analysis using a narrower bandwidth, thereby reducing the potential biases.

Third, our results may be influenced by mortality- or morbidity-related selection in the census and GGS data. Being an unwanted child is generally associated with adverse health outcomes (Nelson et al., 2022) and higher economic vulnerability in adulthood (Hajdu & Hajdu, 2021). Therefore, individuals born during the abortion ban may have died before the census or survey interviews, or they might have been physically or mentally impaired, restricting their participation in the data collection process. If the unwantedness mechanism contributed to further economic and social disadvantages and thus led to a slower transition to adulthood, the underrepresentation of this population would have resulted in a conservative estimate of the effect of the abortion ban on life course trajectories.

Despite its limitations, the present paper has important strengths. We contributed to knowledge about the indirect and the intergenerational effects of population policies by demonstrating how Romania’s abortion ban influenced subsequent generations’ family formation decisions, a topic that was previously underexplored. Additionally, our research underscored the importance of examining gender and socioeconomic differences when investigating the long-term repercussions of pronatalist policies. Lastly, by relying on two complementary data sources and approximating a regression discontinuity design, we accurately identified individuals directly affected by the abortion ban. This method allowed us to effectively minimize the potential biases from confounding factors, thereby strengthening the credibility of our results.

## Supplementary Information

Below is the link to the electronic supplementary material.

Supplementary Material 1

## Data Availability

The Generations and Gender Survey is available upon request at https://www.ggp-i.org/ and Romanian Census data can be accessed at https://international.ipums.org/international/.
