# Supplementary material for: Abortion Ban and the Next Generation’s Family Formation Decisions: Evidence from Romania
Source: Eur J Popul. 2026 Mar 2;42(1):11. doi: 10.1007/s10680-026-09768-8 (PMC13013781; doi:10.1007/s10680-026-09768-8)
Supplement: Supplementary file 1 — Supplementary Material 1 [file 10680_2026_9768_MOESM1_ESM.docx]

SUPPLEMENTARY MATERIAL

**Table S1: The effect of abortion ban on marriage market outcome using Romania Census 2011 (halved bandwidth)**

|  | **Women** | | | | **Men** | | | |
| --- | --- | --- | --- | --- | --- | --- | --- | --- |
|  | Linear RD | BW | Obs. | Mean | Linear RD | BW | Obs. | Mean |
|  |  |  |  |  |  |  |  |  |
| Married | -0.014* | 24 | 63217 | 0.79 | 0.004 | 21 | 55303 | 0.79 |
|  | (0.006) |  |  |  | (0.005) |  |  |  |
| Age differences (men and women) | -0.656*** |  |  |  | -0.156*** |  |  |  |
|  | (0.033) | 11 | 20314 | 3.42 | (0.044) | 18 | 31079 | 3.13 |
| Age at first marriage | 0.289** | 24 | 44051 | 22.64 |  |  |  |  |
|  | (0.028) |  |  |  |  |  |  |  |
|  |  |  |  |  |  |  |  |  |

*Note*: Clustered standard errors in parentheses. + p*<*0.1, * p*<*0.05, ** p*<*0.01. In all specifications, month of birth is included as a control variable. Obs.: Number of observations.

**Table S2:** **The effect of abortion ban on leaving parental home and family formation by gender (GGS – halved bandwith)**

|  | **Women** | | | | **Men** | | | |
| --- | --- | --- | --- | --- | --- | --- | --- | --- |
|  | Linear RD | BW | Obs. | Mean | Linear RD | BW | Obs. | Mean |
|  |  |  |  |  |  |  |  |  |
| Age at leaving home | 0.373 | 15 | 329 | 19.95 | -0.771 | 25 | 449 | 22.7 |
|  | (0.260) |  |  |  | (0.839) |  |  |  |
| Probability of marriage | 0.0197 | 27 | 586 | 0.84 | 0.011 | 21 | 488 | 0.82 |
|  | (0.0315) |  |  |  | (0.062) |  |  |  |
| Age at first marriage | 0.809* | 28 | 498 | 21.4 | -0.650+ | 37 | 633 | 24.84 |
|  | -0.327 |  |  |  | (0.374) |  |  |  |
| Age differences (men-women) | -1.383*** | 25 | 422 | 3.32 | 0.584+ | 25 | 454 | 3.38 |
|  | (0.500) |  |  |  | (0.334) |  |  |  |

*Note*: Clustered standard errors in parentheses. + p*<*0.1, * p*<*0.05, ** p*<*0.01. In all specifications, month of birth is included as a control variable.BW:Bandwidth, Obs.: Number of observations

**Table S3: The effect of abortion ban on leaving parental home and family formation by gender (GGS – adjusted for mother’s education)**

|  | **Women** | | | | **Men** | | | |
| --- | --- | --- | --- | --- | --- | --- | --- | --- |
|  | Linear RD | BW | Obs. | Mean | Linear RD | BW | Obs. | Mean |
|  |  |  |  |  |  |  |  |  |
| Age at leaving home | 1.564** | 35 | 623 | 19.92 | -0.902 | 62 | 961 | 22.63 |
|  | (0.459) |  |  |  | (0.702) |  |  |  |
| Probability of marriage | 0.003 | 53 | 985 | 0.84 | 0.086 | 43 | 896 | 0.83 |
|  | (0.025) |  |  |  | (0.061) |  |  |  |
| Age at first marriage | 0.988** | 54 | 830 | 21.63 | 0.915* | 61 | 946 | 24.86 |
|  | (0.328) |  |  |  | (0.448) |  |  |  |
| Age differences (men-women) | 0.827+ | 51 | 786 | 3.39 | -0.378 | 54 | 878 | 3.30 |
|  | (0.471) |  |  |  | (0.353) |  |  |  |

*Note*: Clustered standard errors in parentheses. +p*<*0.1, * p*<*0.05, ** p*<*0.01. In all specifications, month of birth is included as a control variable.BW:Bandwidth, Obs.: Number of observations

**Table S4: The effect of abortion ban on leaving parental home and family formation by gender (GGS – adjusted for father’s education)**

|  | **Women** | | | | **Men** | | | |
| --- | --- | --- | --- | --- | --- | --- | --- | --- |
|  | Linear RD | BW | Obs. | Mean | Linear RD | BW | Obs. | Mean |
|  |  |  |  |  |  |  |  |  |
| Age at leaving home | 1.772** | 37 | 634 | 20.17 | -0.883 | 65 | 974 | 22.62 |
|  | (0.483) |  |  |  | (0.695) |  |  |  |
| Probability of marriage | 0.003 | 59 | 1019 | 0.83 | 0.069 | 44 | 888 | 0.83 |
|  | (0.027) |  |  |  | (0.058) |  |  |  |
| Age at first marriage | 1.150** | 65 | 927 | 21.48 | -0.808+ | 59 | 890 | 24.86 |
|  | (0.322) |  |  |  | (0.447) |  |  |  |
| Age differences (men-women) | -0.892+ | 48 | 718 | 3.51 | -0.150 | 51 | 823 | 3.39 |
|  | (0.518) |  |  |  | (0.358) |  |  |  |

*Note*: Clustered standard errors in parentheses. + p*<*0.1, * p*<*0.05, ** p*<*0.01. In all specifications, month of birth is included as a control variable.BW:Bandwidth, Obs.: Number of observations
